# Aquaporins modulate the cold response of *Haemaphysalis longicornis* via changes in gene and protein expression of fatty acids

**DOI:** 10.1186/s13071-025-06718-x

**Published:** 2025-02-24

**Authors:** Han Wang, Ruwei Bai, Tingwei Pei, Jianglei Meng, Chuks F. Nwanade, Yuchao Zhang, Xiujie Liang, Yunsheng Tang, Jingze Liu, Zhijun Yu

**Affiliations:** 1 Hebei Key Laboratory of Animal Physiology, Biochemistry and Molecular Biology, Hebei Collaborative Innovation Center for Eco-Environment, Hebei Research Center of the Basic Discipline of Cell Biology, Ministry of Education Key Laboratory of Molecular and Cellular Biology, College of Life Sciences, Hebei Normal University, Shijiazhuang, 050024 China; 2 Guangdong Key Laboratory of Animal Conservation and Resource Utilization, Guangdong Public Laboratory of Wild Animal Conservation and Utilization, Institute of Zoology, Guangdong Academy of Sciences, Guangzhou, 510260 China

**Keywords:** *Haemaphysalis longicornis*, Aquaporin, Cold response, Water balance, Fatty acid metabolism

## Abstract

**Background:**

As ectotherms that spend most of their life in the environment (off-host), ticks face challenges in maintaining water balance, and some species must cope with severe low winter temperatures. Aquaporins (AQPs) are essential membrane proteins that enhance cold tolerance in many animals by regulating homeostatic processes. However, the dynamic expressions and involvement of aquaporins in the cold stress of ticks remain unclear.

**Methods:**

In the present study, three AQP genes, *HlAQP2*, *HlAQP3*, and *HlAQP5*, belonging to the major intrinsic protein (MIP) superfamily, were characterized from the important vector tick *Haemaphysalis longicornis*. Then, multiple bioinformatics analyses were performed. Quantitative real-time PCR (qPCR) was used to detect different expressions of *H. longicornis* genes under different cold treatment conditions. RNA interference was used to explore the relationship between AQP and the cold response of *H. longicornis*. Additionally, proteomic and transcriptomic analyses were used to investigate the mechanisms underlying the effects of AQPs on cold response in ticks.

**Results:**

The amino acid sequence of AQPs shows high homology in Ixodida, with *HlAQP2* and *HlAQP5* proteins comprising two asparagine-proline-alanine (NPA) motifs, whereas *HlAQP3* protein was featured by glycerol facilitator GlpF channel. The spatiotemporal expression of AQPs in *H. longicornis* varied significantly after low temperature treatment, and different expression patterns were observed over prolonged exposure periods. RNAi knockdown of AQPs significantly increased tick mortality after treatment at a sublethal temperature of − 14 °C for 2 h. Proteomic and transcriptomic analysis revealed that the differentially expressed genes and proteins caused by the knockdown of AQPs are mainly enriched in the fatty acid metabolism pathway.

**Conclusions:**

The above results indicated that AQPs could regulate tick cold response by modulating water balance and fatty acid metabolism.

**Graphical Abstract:**

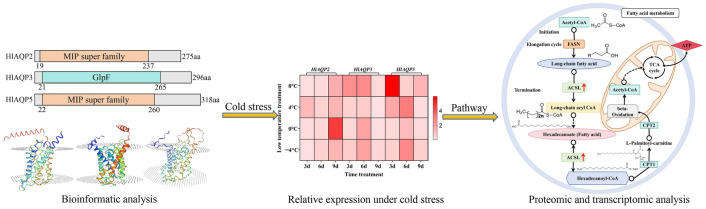

**Supplementary Information:**

The online version contains supplementary material available at 10.1186/s13071-025-06718-x.

## Background

Ticks are important blood-sucking arthropod ectoparasites of wild or domesticated animals, which pose severe health risks to humans and animals by transmitting a great diversity of pathogens including viruses, rickettsiae, helminths, bacteria, and protozoans [1, 2]. As ectotherms, ticks (ixodid ticks) spend most of their lives in the environment (on-host or off-host), and their development and survival are highly dependent on environmental conditions [3]. Temperature is one of the main environmental factors that can greatly affect the metabolism, water balance, and reproduction of ticks and further limit their distribution and population expansion [4]. The cold, dry conditions of temperate winters are usually the major challenges for tick survival, and previous research has demonstrated that ticks display varying abilities to tolerate extreme temperatures [5]. Ticks have developed multiple behavioral and physiological overwintering strategies. They can escape low temperatures by finding suitable sheltering microhabitats before the onset of winter [6] and overwinter in the deciduous layer or duff layer of soil to ensure high survival rates [7]. Some ticks enter a diapause (dormancy) or quiescence state to survive the lethal temperatures or harsh environments [8]. Simultaneously, ticks modulate the production and accumulation of small molecular cryoprotectants and antifreeze proteins to enhance cold tolerance [9], and cold acclimation has also been demonstrated to increase tick survival [10].

The regulation of aquaporins (AQPs) is a key process of living organisms in enhancing cold tolerance by regulating essential homeostatic processes [11]. AQPs are intrinsic transmembrane proteins belonging to the Major Intrinsic Protein (MIP) superfamily and are ubiquitous throughout plants and animals [12]. AQPs can form pores for the rapid transcellular movement of water and some small molecules, such as glycerol, or volatile substances [13]. It has been demonstrated that AQPs play diverse roles under different abiotic stresses including dehydration, salinity, and cold stress [14] as well as in various physiological processes such as neural signal transduction, skin moisturization, fat metabolism, and exocrine gland secretion [15, 16]. In insects, the involvement of the aquaporin gene family in response to low temperatures and desiccation stress has been characterized and functionally validated. For instance, in the larvae of the Chinese white pine beetle *Dendroctonus armandi*, *DaAqps* were found downregulated with decreasing temperatures and long-term exposure to cold conditions, which suggested that AQPs may be coordinated to tolerate low-temperature stress conditions [17]. Overwintering larvae of the goldenrod gall fly, *Eurosta solidaginis*, are both freeze tolerant and desiccation resistant. Larvae acquire freeze tolerance during the autumn, in part through the accumulation of glycerol and sorbitol. The aquaporins enable the diapausing larvae to survive at − 50 ºC [18]. In the Antarctic midge *Belgica antarctica*, some AQPs were found upregulated to increase water loss during dehydration; others were downregulated to conserve water in specific tissues [19].

The tick *Haemaphysalis longicornis* is widely distributed in East Asia, New Zealand, and Australia, with a recently established population in the eastern USA [20]. It can transmit many pathogens including *Borrelia burgdorferi*, *Anaplasma phagocytophilum*, *Theileria* spp, *Babesia* spp, severe fever with thrombocytopenia syndrome virus (SFTSV), spotted fever group rickettsiae (SFGR), tick-borne encephalitis virus, and Alongshan virus (ALSV) [21, 22]. Numerous genes and proteins including small heat shock proteins [23], superoxide dismutase [24], and heat shock protein 70/90 and tubulins [25] have been characterized and associated with tick response to low-temperature stress. Nevertheless, knowledge about the versatile functions and regulation of tick AQPs in response to cold stress remains meager.

In the present study, three AQPs were characterized in the tick *H. longicornis*, followed by the evaluation of the spatiotemporal expression of AQPs during cold response using quantitative real-time PCR (qPCR) and functionally confirmed by RNA interference. Furthermore, the underlying mechanisms involved in the regulation of AQPs were determined by integrative proteomic and transcriptomic analysis, hoping to shed light on the molecular regulatory mechanisms involved in the cold response of ticks.

## Methods

### Tick rearing and cold treatment

The unfed *H. longicornis* adults were collected by flag-dragging from the vegetation in the Xiaowutai National Nature Reserve Area (39°50′, 114°47′), Hebei province, China. They were brought back to the laboratory and maintained in an incubator [26 ± 1 °C, 75 ± 5% relative humidity (RH), 16:8 h light/dark photoperiod] during non-feeding periods. For feeding, ticks were put into the cloth bags attached to the ears of domestic rabbits, as described previously. Second-generation adult female ticks 2 weeks post-molt were randomly selected and used for subsequent analysis. For cold treatment, groups of 20 unfed ticks were kept in a digital thermostatic bath at different temperatures (− 4 °C, 0 °C, 4 °C, and 8 °C) for periods of 3, 6, and 9 days, respectively. Ticks maintained at 26 °C served as the control group, and each treatment temperature was replicated three times. All experiments involving animals were conducted with the approval of the Animal Ethics Committee of Hebei Normal University (protocol no. IACUC-209216).

### RNA extract and cDNA synthesis

Total RNA was extracted using TransZol UP (Invitrogen, Carlsbad, CA, USA) following the manufacturer’s instructions. RNA quantification was determined using a NanoDrop ND-2000 Spectrophotometer (Thermo Fisher Scientific, Waltham, MA, USA), with A260/A280 ratios generally > 2.0, and the RNA quality was confirmed by 1% agarose gel electrophoresis. Reverse transcription was performed to synthesize complementary DNA (cDNA) using the TransScript One-Step gDNA Removal and cDNA Synthesis SuperMix (Thermo Scientific), following the manufacturer's protocol, with the polymerase chain reaction (PCR) conditions set as follows: 42 °C for 15 min, followed by 85 °C for 5 s. Subsequently, primers (Table S1) were designed using Primer Premier version 5.0 based on the genomic sequences (Bio-Project PRJNA668644) [26]. PCR conditions including an initial denaturation at 94 °C for 2 min and 40 cycles at 94 °C for 30 s, 30 s at the melting temperature Tm of 54–59 °C (based on different sequences), and 30 s at 72 °C followed by a final extension at 72 °C for 10 min. The amplified fragments were verified and separated on a 1% agarose gel. Bands with expected sizes were excised and purified by EG101-01 EasyPure Quick Gel Extraction Kit (TransGen, Beijing, China) for sequencing.

### Bioinformatic analysis

The sequence alignment and identity were performed using DNAMAN with a similarity > 90%. Homology comparison was performed at NCBI using Blast (https://blast.ncbi.nlm.nih.gov/Blast.cgi) [27]. The genes were converted into amino acid sequences using the Expasy website (https://www.expasy.org/) and DNAMAN, and the physicochemical properties were predicted using ExPASy-ProtPram (http://web.expasy.org/protparam). The relative molecular weight and base composition were analyzed by the Expasy website and BioEdit. The structural domains were constructed using CD-search in NCBI (https://www.ncbi.nlm.nih.gov/Structure/cdd/wrpsb.cgi), and the transmembrane regions were predicted using TMHMM v.2.0 (http://www.cbs.dtu.dk/services/TMHMM/). Subcellular localization was performed with ProtComp 9.0 (http://www.softberry.com/berry.phtml?topic=protcompan&group=programs&subgroup=proloc). The secondary structure was predicted via the SOMPA (https://npsa-pbil.ibcp.fr/cgi-bin/npsa_automat.pl?page=npsa_sopma.html) website. Swiss-Model (https://swissmodel.expasy.org/) predicts tertiary structure. Evolutionary analyses were conducted in MEGA11 using the neighbor-joining method [28].

### Relative expression of AQPs in *H. longicornis* under cold stress

The expression dynamics of AQPs during the cold response of *H. longicornis* were determined using qPCR. To standardize the relative expression of the target genes, several commonly used reference genes (GAPDH, tubulin, and β-actin) in *H. longicornis* were preliminarily tested under cold treatment for different time periods; finally, β-actin was chosen to serve as the reference gene for its relatively stable expression during cold treatment (Fig. S1). Total RNA was extracted, and cDNA was synthesized as described above. The qPCR reaction was performed on a 96-well optical reaction plate (Roche, USA) using a Roche LightCycler 480 PCR system. The conditions were optimized as follows: 94 °C for 30 s, 94 °C for 40 cycles of 5 s, 60 °C, 95 °C for 1 min, 55 °C for 30 s, and 95 °C for 30 s, followed by a melting curve analysis. The experiments were repeated three times. The relative expression was calculated using the 2^−ΔΔCt^ method, and data analysis was performed by Graphpad Prism 8.0 software (Graphpad, California, USA).

### Cold tolerance of ticks after RNAi

For dsRNA synthesis, primers were designed using Primer Premier 5 (Table S1), and the T7 promoter sequence (5' TAATACGACTCACTATAGG 3') was added at the 5' end of each primer. Before injection, a group of ticks was sequentially surface sterilized with ddH_2_O, hydrogen peroxide, and ddH_2_O. One microliter of dsRNA was injected through the third and fourth coxa using Microliter™ Syringes (Hamilton, Nevada, USA), and the control group was injected with 8000 ng dsRNA of *gfp* (GenBank accession no. KX247384). Each experiment was repeated three times. The ticks were then placed in the environmental incubator (26 ± 1 °C, RH 75 ± 5%, 16 h L:8 h D) for 24 h to recover. After confirmation of the knockdown of the target genes by qPCR as described above, the *H. longicornis* ticks were exposed to a sublethal temperature of − 14 °C for 2 h [29]. Then, the mortality was calculated with ticks considered dead if they remained inactive after CO_2_ stimulation.

### Data independent acquisition (DIA) proteomic analysis

After confirming the knockdown of the AQPs, 20 ticks were used to extract proteins using RIPA:PMSF lysis buffer (100:1), with bicinchoninic acid (BCA) Protein Assay Kit (CWBIO, Jiangsu, China) used to determine the amount of protein. The experiments were repeated three times. Then, the proteins were freeze-dried into powder and reduced with dithiothreitol (10 mM) and alkylated with iodoacetamide (20 mM). Subsequently, they were digested with trypsin and analyzed using DIA-mass spectrometry. Enzymatically digested peptide samples were reconstituted in mass spectrometry-grade water containing 0.1% formic acid (FA), and the appropriate proportion of iRT was added. Quantitative proteomic assays were carried out by Vanquish Neo UHPLC liquid chromatography and Orbitrap Exploris 480 mass spectrometry using the DIA mode (Table S2).

DIA data were retrieved and analyzed using Spectronaut (v.18.0, Biognosys, Switzerland) quantitative proteome analysis software. Protein and peptide FDR < 1% and Q value < 0.05, others were default parameters, and the database used protein sequences generated from the transcriptome of *H. longicornis* assembled in our laboratory (NCBI accession no. GHLT00000000). Differential proteins were screened according to the criteria of 1.5-fold up- or downregulation and converted to UniProtKB numbers for subsequent analysis on the Uniprot website. GO enrichment analysis and KEGG pathway enrichment analysis were performed using the Wukong proteomics data analysis cloud platform (https://www.omicsolution.org/wkomics/main/). The proteomic raw data were deposited at the freely available public data platform ProteomeXchange Consortium (http://proteomecentral.proteomexchange.org) with the data set identifier PXD058058 [30].

### Transcriptomic sequencing

After confirming the knockdown of AQPs, total RNA was extracted using the TRIzol reagent as described above, with 20 ticks in each group, repeated three times. For library construction, poly A-containing mRNA in total RNA was converted into a cDNA library using oligo-dT magnetic beads. Then, a single “A” base to the 3' end was added for the ligation of Illumina adapters. Finally, the products were purified and enriched with PCR to create the final double-stranded cDNA library using NEBNext® Ultra™ RNA Library Prep Kit for Illumina^®^. Sequencing was carried out using the Illumina Novaseq 6000 sequencing platform. Then, the data were filtered, and the quality-controlled clean reads were used for reference genome comparison and gene expression (FPKM) calculation. Clean reads were used for reference genome comparison and gene expression (FPKM) calculation. Differentially expressed genes (DEGs) were analyzed using DESeq2 software, with the criteria set as q value < 0.05, fold change > 2, or fold change < 0.5. Gene set enrichment analysis was performed using GSEA software. GO and KEGG enrichment analyses of DEGs were performed using the hypergeometric distribution algorithm to screen for significantly enriched functional entries.

### Statistical analysis

Statistical analyses were performed using GraphPad Prism 8.0 (GraphPad Software, Inc., San Diego, CA, USA). The data follow normality, and t-test and one-way ANOVA were used to compare the relative expressions and mortality rates, with *p* < 0.05 considered statistically significant. Results were presented as mean ± standard deviation.

## Results

### Characterization and bioinformatic analysis of AQPs

Three AQP genes were identified from the genome of *H. longicornis*, named *HlAQP2*, *HlAQP3*, and *HlAQP5* based on the sequence homology and physicochemical properties (Table 1). The sequences were submitted to GenBank under accession numbers OR754283, OR754284, and OR754285. NCBI Conserved Domain (CD) search found that *HlAQP2* and *HlAQP5* belong to the Membrane Intrinsic Proteins (MIPs) superfamily with two asparagine-proline-alanine (NPA) motifs, whereas *HlAQP3* protein was featured by glycerol facilitator GlpF channel (Fig. S2A). The *HlAQP2, HlAQP3*, and *HlAQP5* proteins were dominant with alpha helix and random coil (Fig. S2B), and the GMQE values estimated were 0.92, 0.89, and 0.86, respectively (Fig. S2C). The proteins of *HlAQP2*, *HlAQP3*, and *HlAQP5* were characterized with six, three, and five transmembrane structural domains, respectively (Fig. S2D). Subcellular localization analysis showed most *HlAQP2*, *HlAQP3*, and *HlAQP5* proteins were located in the cell membrane with 66.7%, 47.80%, and 55.60%, respectively. They are stable hydrophobic proteins (Fig. S3D-F), with *HlAQP2* protein containing 26 phosphate sites, *HlAQP3* containing 40 phosphate sites, and *HlAQP5* containing 24 phosphate sites. The phylogenetic analysis found that the AQPs in *H. longicornis* belong to the same branch as that from *Dermacentor silvarum*, *Dermacentor andersoni*, *Rhipicephalus sanguineus*, *Rhipicephalus microplus*, and *Ixodes scapularis* (Fig. S3A–C).

**Table 1:** Predicted characteristics of the aquaporins in *Haemaphysalis longicornis*

Gene name	CDS (bp)	Amino acid number (aa)	Atom number	Molecular mass (kDa)	PI	Hydrophobicity index	Instability index	Aliphatic index
*HlAQP2* (OR754283)	828	275	4069	28.77258	6.19	0.610	27.95	102.8
*HlAQP3* (OR754284)	891	296	4390	31.12313	8.75	0.502	32.00	100.81
*HlAQP5* (OR754285)	975	318	4875	34.76626	6.82	0.374	27.30	93.52

CDS, coding sequence; PI, isoelectric point

### Relative expression of AQPs in *H. longicornis* under cold stress

**Fig. 1:**
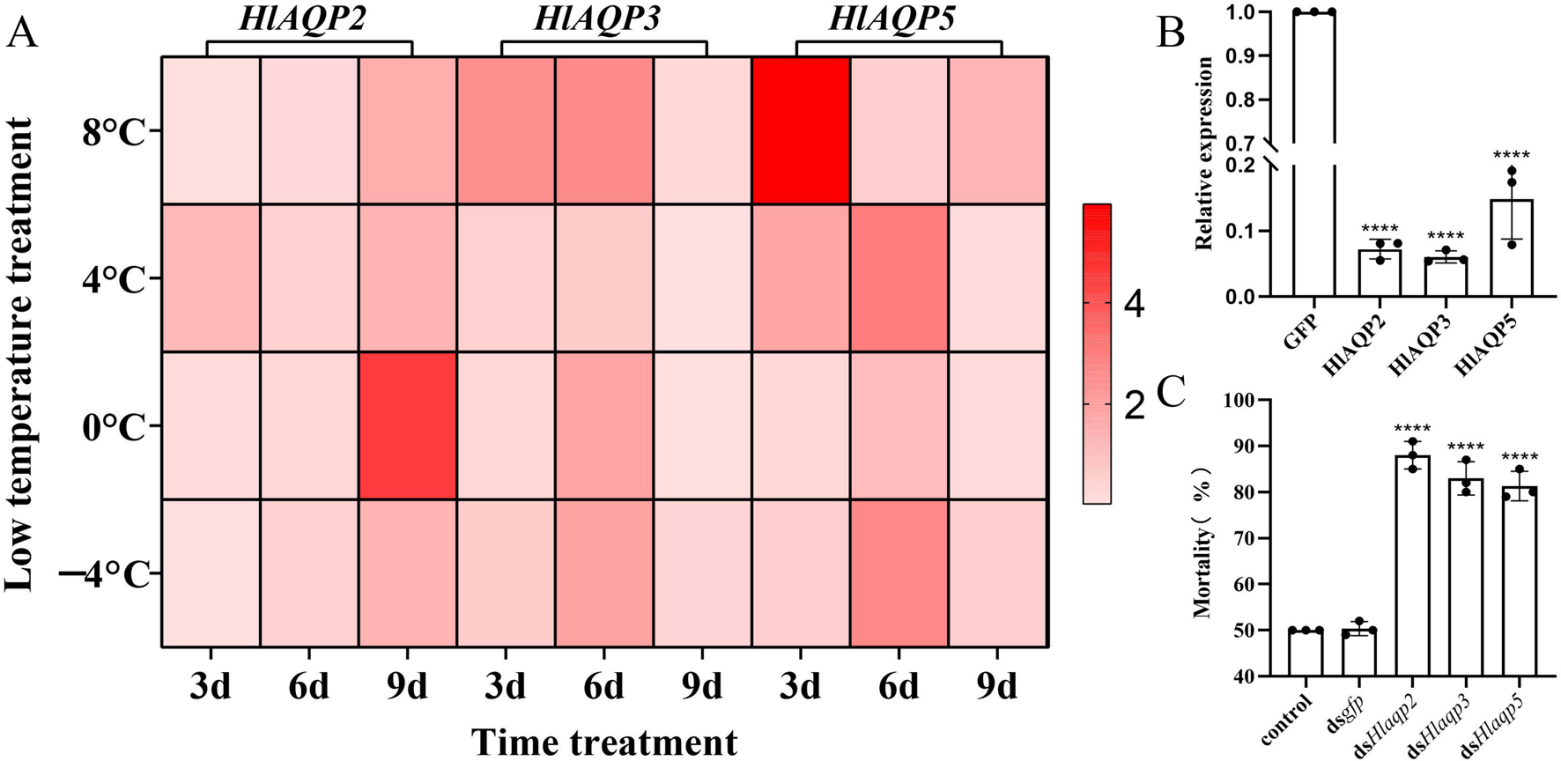
The spatiotemperal expression and function evaluation of aquaporins in *Haemphysalis longicornis* under different cold treatment. **A** The relative expression of AQP mRNA of *H. longicornis* at different temperatures and different times. **B** Real-time mRNA expression efficiency of *H. longicornis* after interference with AQP gene (****p < 0.0001). **C** Mortality of *H. longicornis* at semi-lethal temperature after interference with AQP genes (****p < 0.0001)

The relative expression of AQPs showed different expression trends in *H. longicornis* when treated at − 4 °C, 0 °C, 4 °C, and 8 °C for 3, 6, and 9 days. When the *H. longicornis* ticks were treated at − 4 °C, 0 °C, and 8 ℃, the *HlAQP2* showed a decreasing trend (*p* < 0.00001) in the first 3 days and then increased (*p* < 0.001). No significant change in *HlAQP2* was observed when treated at 4 ℃ for 3 days, which followed a decreasing trend and reached the lowest value (*p* < 0.01) on the 6th day and then increased (*p* < 0.01) (Fig. 1A).

*HlAQP3* showed a decreasing trend in the first 3 days when treated at 0 °C and 4 °C (*p* < 0.001), and increased thereafter (*p* < 0.05), but decreased on day 6 and reached the lowest value on day 9 (*p* < 0.00001). After treatment at − 4 °C, no significant change was observed for *HlAQP3* in the first 3 days, reaching a peak on day 6 (*p* < 0.05) and then decreasing (*p* < 0.01). When treated at 8 °C, the expression of *HlAQP3* increased on the 3rd day (*p* < 0.01) and started to decrease on the 6th day until reaching the lowest value on day 9 (*p* < 0.00001) (Fig. 1A).

The expression of *HlAQP5* showed a decreasing trend (*p* < 0.001) for the first 3 days, reached the peak on the 6th day, and then decreased to the lowest value on day 9 (*p* < 0.01) when treated at − 4 °C and 0 °C. After treatment at 4 °C, it showed an increasing trend (*p* < 0.001) for the first 6 days and then decreased to the lowest value by day 9 (*p* < 0.00001). When treated at 8 °C, *HlAQP5* showed an increasing trend for the first 3 days (*p* < 0.001), decreased to the lowest value on day 6 (*p* < 0.001), and showed a slight increase on day 9 (*p* < 0.01) (Fig. 1A).

### Function of AQPs contributing to the cold tolerance of *H. longicornis*

The expression of AQPs was successfully knocked down using RNAi, with the knockdown efficiency of *HlAQP2*, *HlAQP3*, and *HlAQP5* reaching 93%, 94%, and 86%, respectively (Fig. 1B). Then, the ticks were treated at a sublethal temperature (− 14 °C) for 2 h, and a significant increase in mortality was found in *H. longicornis* after knockdown of the AQPs (*p* < 0.0001) (Fig. 1C).

### Identification of proteins modulated by AQPs during cold stress of *H. longicornis*

Protein profiling of ticks after AQP knockdown showed a total of 3226 intersecting proteins among the four groups of ds*gfp*, ds*HlAQP2*, ds*HlAQP3*, and ds*HlAQP5*, as demonstrated by the Venn diagram (Fig. S4A). In detail, the differentially expressed proteins in *HlAQP2* dsRNA-injected *H. longicornis* were mainly enriched in sulfation, protein import into the nucleus, positive regulation of GTPase activity, and mRNA processing in the biological process (BP); the nucleus, integral component of membrane, and cytoplasm in cellular component (CC); and ATP binding and metal ion binding in molecular function (MF) (Fig. S4B). The differentially expressed proteins in the *HlAQP3* dsRNA-injected group were mainly enriched in signal transduction, mRNA processing, and carbohydrate metabolic process in BP and the integral component of membrane in CC, metal ion binding and ATP binding in MF. The differentially expressed proteins in the *HlAQP5* dsRNA-injected group were mainly enriched in translation in BP, an integral component of the membrane in CC, and RNA binding in MF (Fig. S4B, Tables S3-5).

KEGG enrichment showed that, after the knockdown of *HlAQP2*, *HlAQP3*, or *HlAQP5*, most of the differentially expressed proteins were mainly enriched in metabolic pathways (Fig. S4C). The differentially expressed proteins were found mainly enriched in protein processing in purine metabolism, nucleotide metabolism, and ATP-dependent chromatin remodeling in the ds-*HlAQP2* injection group. After the knockdown of *HlAQP3*, the differentially expressed proteins were found mainly enriched in fatty acid-related pathways including fatty acid biosynthesis, degradation, and metabolism, as well as peroxisome, whereas after the knockdown of *HlAQP5*, the differentially expressed proteins in ATP-dependent chromatin remodeling pathways were found. The fatty acid-related pathways also changed in the ds-*HlAQP5* injection group (Fig. S4C).

The proteomic analysis most significantly affects metabolism-related signaling pathways. The differentially expressed proteins include long-chain fatty acid-CoA ligase (ACSL), adenosine kinase (ADK), and chromatin complex subunit BAP18, which are involved in fatty acid biosynthesis, purine metabolism, and ATP-dependent chromatin remodeling. With the involvement of these signaling pathways, many proteins related to metabolic processes are also affected.

### Gene expression modulated by AQPs during cold stress of *H. longicornis*

**Fig. 2:**
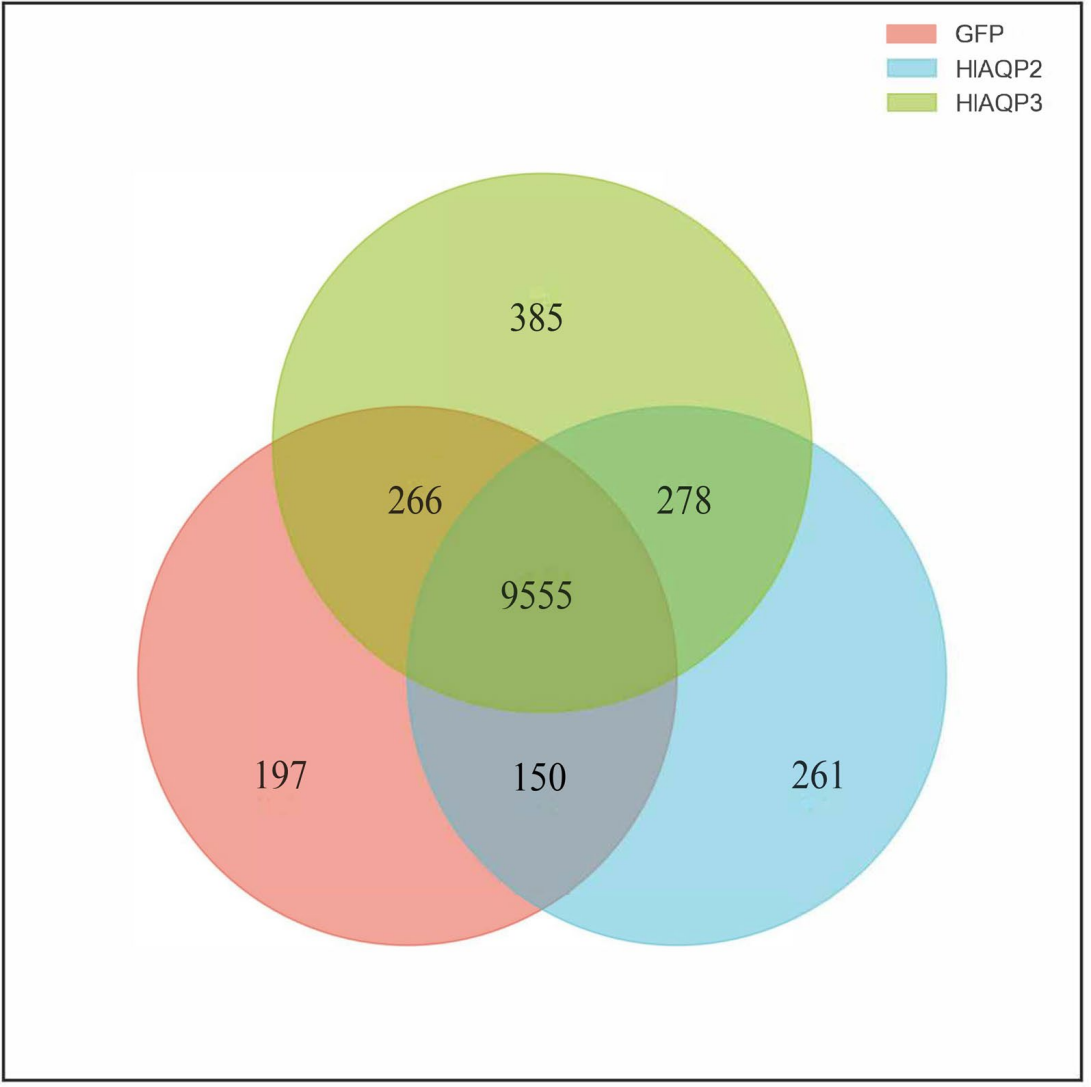
Venn diagram shows the overlap and specificity of gene identification between different treatment groups; 9555 intersection genes were identified after *HlAQP2* and *HlAQP3* knockdown in *Haemaphysalis longicornis*

**Fig. 3:**
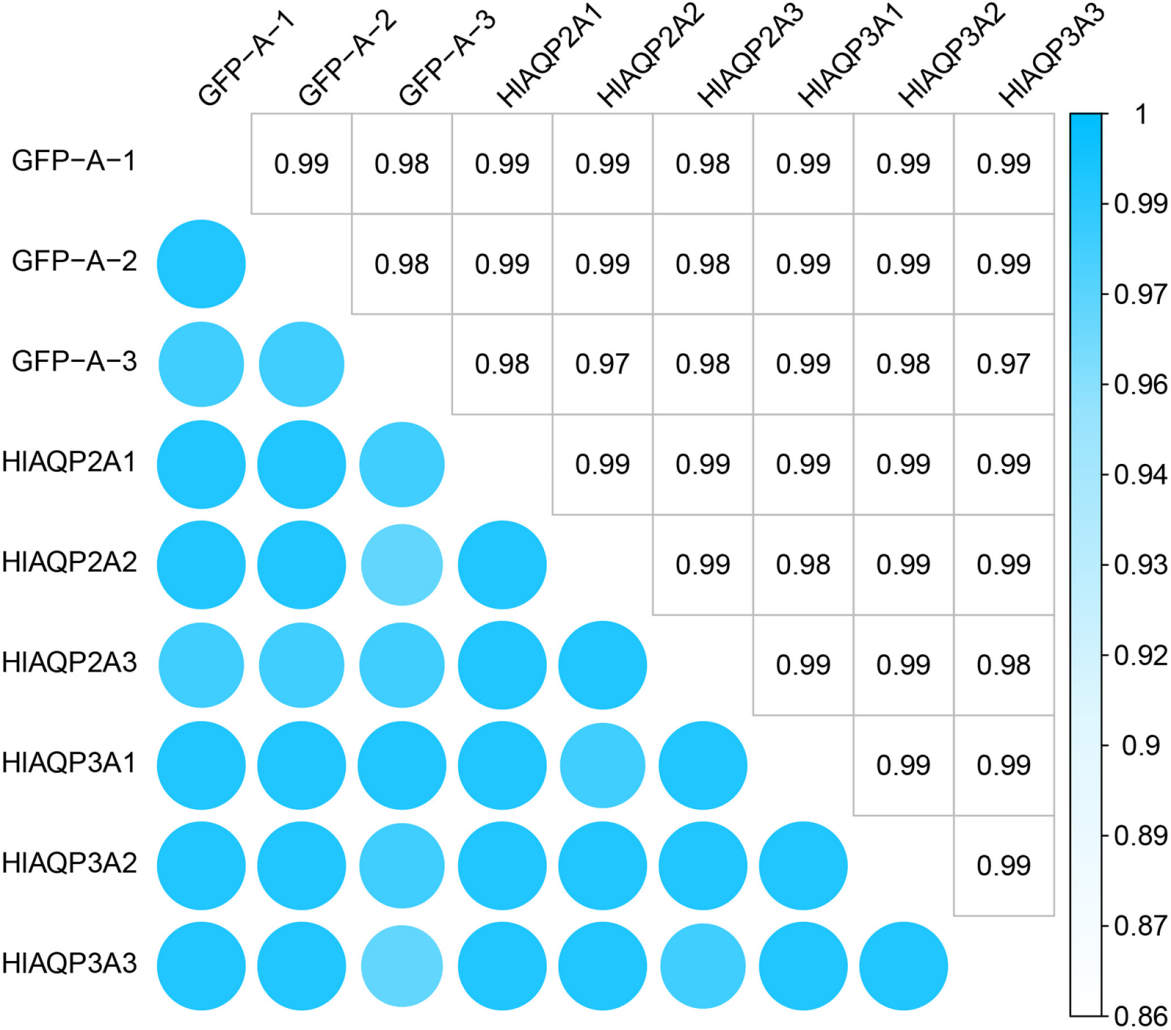
Sample-to-Sample distances heatmap analysis after *HlAQP2* and *HlAQP3* knockdown

After RNAi, transcriptomic sequencing was carried out to evaluate the gene expression modulated by AQPs during cold stress of *H. longicornis*. The overall distribution of differentially expressed genes was visualized by plotting the Venn diagram and a Sample-to-Sample distances heatmap. A total of 9555 raw reads were shared by the ds-*HlAQP2* injection group, ds-*HlAQP3* injection group, and control group (Fig. 2). Significant correlations were observed between *HlAQP2*-dsRNA and *HlAQP3*-dsRNA injection groups compared with the ds-*gfp* injection group (Fig. 3). GO enrichment showed that the differentially expressed genes in ds-*HlAQP2* injection group were mainly enriched in proteolysis involved in the protein catabolic process in BP, extracellular region in CC, and iron ion binding and heme binding in MF (Fig. S5A, Table S6). GO enrichment of the ds-*HlAQP3* injection group revealed that the differentially expressed genes were mainly associated with functions such as negative regulation of peptidase activity and response to estrogen in BP, extracellular space in CC, and signaling receptor binding in MF (Fig. S5B; Table S7).

**Fig. 4:**
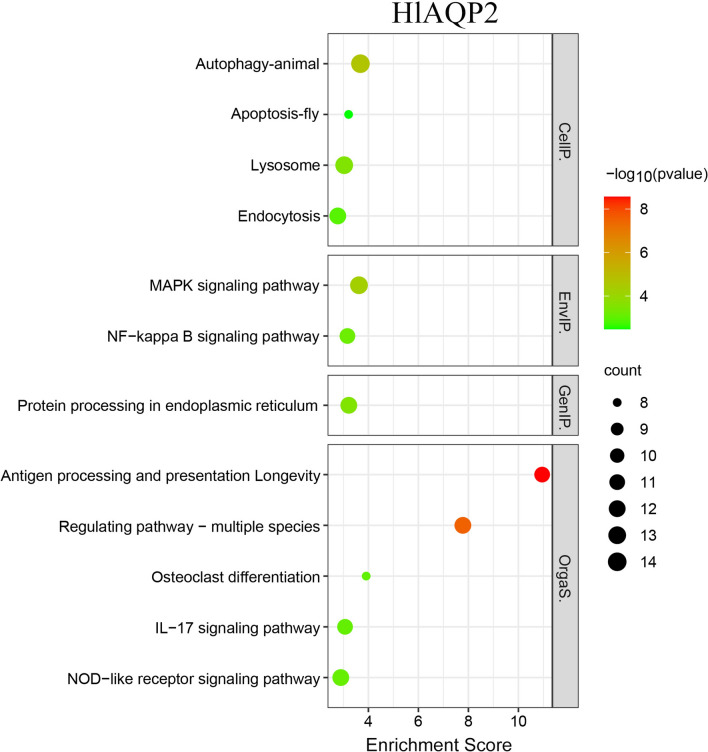
KEGG enrichment analysis of *HlAQP2* differentially expressed genes after RNAi in *Haemaphysalis longicornis*

**Fig. 5:**
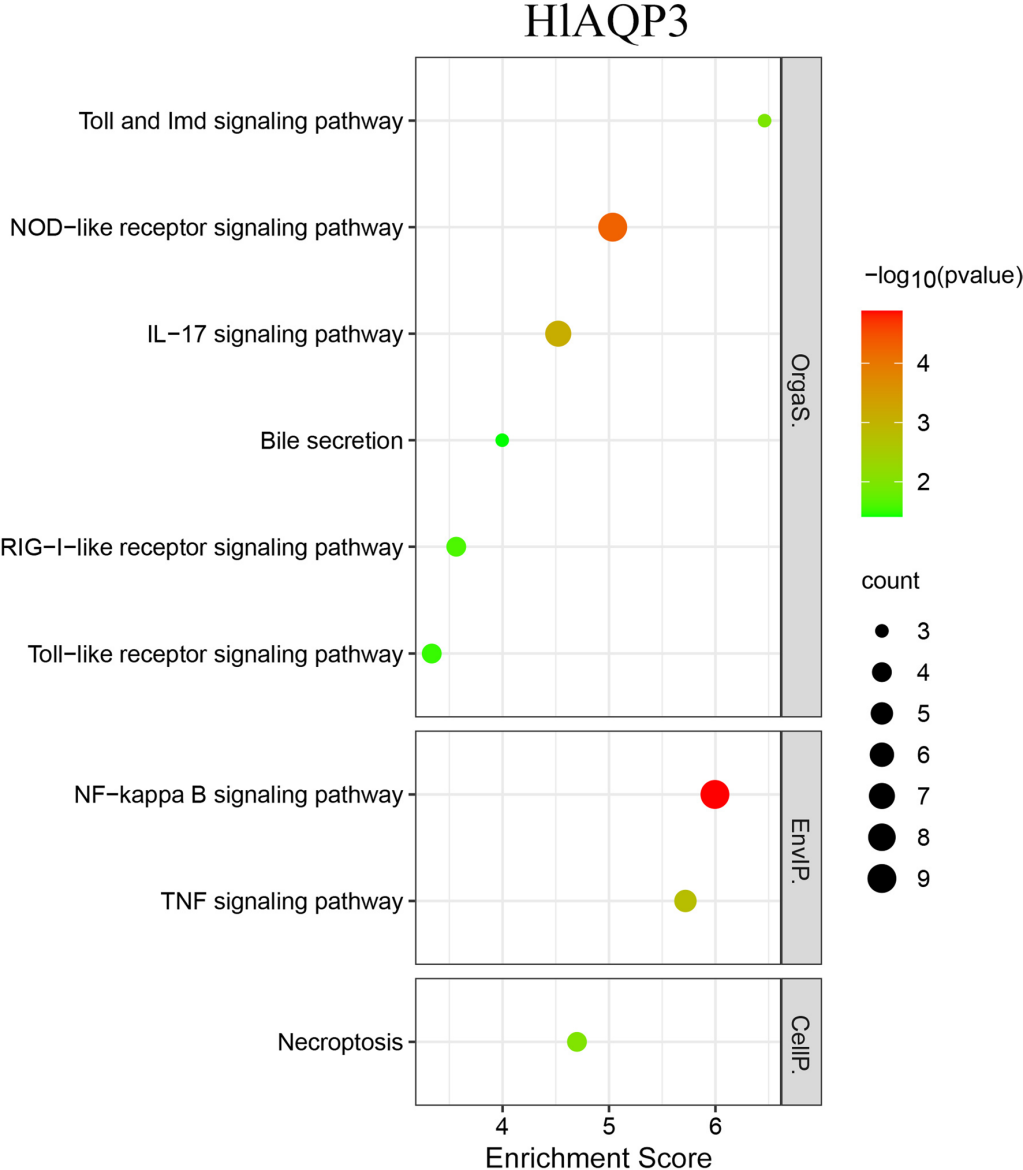
KEGG enrichment analysis of *HlAQP3* differentially expressed genes after RNAi in *Haemaphysalis longicornis*

KEGG enrichment analysis showed that after knockdown of *HlAQP2*, the differentially expressed genes were mainly enriched to autophagy-animal, lysosome, MAPK signaling pathway, NF-kappa B signaling pathway, and antigen processing and presentation (Fig. 4). After knockdown of *HlAQP3*, KEGG enrichment analysis showed that the differentially expressed genes were mainly enriched to NF-kappa B signaling pathway, NOD-like receptor signaling pathway, and Toll-like receptor signaling pathway (Fig. 5).

The transcriptomic analysis found that most of the differentially expressed genes in metabolic pathways and signal transduction were affected, which included insulin, cathepsin L, intracellular cholesterol transporter 1 (Niemann-Pick C1, NPC1), and TNF receptor-associated factor 6 (TRAF6), as well as genes involved in the autophagy pathway, lysosome pathway, NF-kappa B signaling pathway, and fatty acid metabolism pathway.

## Discussion

AQPs are mainly localized in the plasma membrane in many cell types, which are essential for maintaining water balance and associated with multiple physiological processes [31]. In the present study, three AQP genes were cloned and identified in *H. longicornis*, and their physicochemical properties were predicted by bioinformatic analysis. Furthermore, the spatiotemporal expressions during the cold response were determined, with the functions contributing to the cold stress of ticks verified by RNAi, and the underlying molecular mechanism and regulation of AQPs were explored through integrative proteomic and transcriptomic analysis.

The *HlAQP2* and *HlAQP5* proteins belong to the MIP superfamily and were characterized by two asparagine-proline-alanine (NPA) amino acid motifs; they can form six and five transmembrane structural domains, respectively. A similar structure was also found in *IsAQP1* from *Ixodes scapularis* [32] and *SlAQP2* and *SlAQP3* from *Spodoptera littoralis* [33]. The NPA motifs are usually in the narrow central constriction of the channel formed by AOPs and are essential for selective and efficient water passage [12]. The NPA motifs found in both *HlAQP2* and *HlAQP5* proteins imply their important roles in regulating the water balance of *H. longicornis*. The GlpF motif is a highly selective transmembrane channel that conducts glycerol and certain other small uncharged organic molecules [34]. *HlAQP*3 was featured by the glycerol facilitator GlpF and three transmembrane structural domains, indicating its compensatory function with *HlAQP2* and *HlAQP5*.

Temperature is one of the key abiotic stressors faced by arthropods, and many insects cope with low temperatures through a variety of adaptive mechanisms. For example, larvae of the American dog tick are able to survive low temperatures by adapting physiological mechanisms [6], while beetles exhibit remarkable low-temperature resilience, with strategies that include reproductive arrest and lifespan extension [35]. In addition, body color darkening plasticity in flies is strongly correlated with cold temperature adaptation, and there are significant differences in the ability of different flies *Drosophila* to adapt at low temperatures [36]. Springtails, on the other hand, enhance cold tolerance by regulating cell membrane fluidity and enhancing antioxidant capacity [37]. Among these adaptive mechanisms, AQPs may play an important role in low-temperature acclimation of *H. longicornis*, and AQP helps to maintain the integrity of cell membranes by regulating intracellular water balance and osmotic pressure, thus enhancing the survival of *H. longicornis* in low-temperature environments.

The regulation of water content by AQPs is closely related to the cold tolerance of insects [38]. In the Eastern Grass Veneer moth *Agriphila aeneociliella*, *AaAQP1*, *AaAQP3*, and *AaAQP6* were significantly upregulated in larvae after low temperature exposure, indicating their involvement in its cold tolerance [11]. *EsAQP1* of *E. solidaginis* has been demonstrated to cryoprotect the brain from damage associated with water imbalance [39]. Dynamic expressions of *AsAQP2* and *AsAQP4* characterized from *Anopheles sinensis* were observed when exposed to low temperature. The *AsAQP4* expression level increased and survival time reduced at the freezing point (0 °C). *AsAQP2* and *AsAQP4* expression levels decreased and survival time was significantly extended when exposed to low temperature (8 °C) [21]. In *H. longicornis*, it has been demonstrated that cold acclimation can trigger a multifaceted response, including water redistribution and cryoprotectant accumulation [21]. This facilitates solute and water movement, augmenting hydraulic conductivity and cellular freeze resistance. In the present study, the expressions of *HlAQP3* and *HlAQP5* decreased at − 4 °C and 0 °C for 3 days and then increased, followed by another decrease. A similar phenomenon was also observed in the Chinese white pine beetle *D. armandi*; the aquaporins were downregulated with decreasing temperatures and long-term exposure to cold conditions, and when the lowest temperatures were reached, the levels were immediately upregulated [17]. The early low expression of AQPs in *H. longicornis* indicates the preparation for cold stress, and the subsequent increase suggests the important involvement and function during the maintenance of cold tolerance. In addition, after the knockdown of *HlAQP2*, *HlAQP3*, and *HlAQP5*, the mortality of *H. longicornis* increased significantly. These results indicate that the AQPs play important roles in the cold tolerance of *H. longicornis*. The transcriptional response of AQPs depends on the severity and duration of cold stress [40].

After knockdown of the AQPs of *H. longicornis*, proteomic analysis revealed that upregulation of long-chain fatty acid-CoA ligase (ACSL), adenosine kinase (ADK), and chromatin complex subunit BAP18 enhanced fatty acid biosynthesis, purine metabolism, and ATP-dependent chromatin remodeling, respectively. Fatty acid metabolism is related to energy storage, enzyme reaction rate, molecular transport, and diffusion, which are essential for insect growth, development, and physiological adaptation [41]. ACSL plays a crucial role in fatty acid (FA) metabolism, which is present in various organisms and is involved in energy homeostasis, fatty acid transport, post-translational modification of proteins, signaling, and cell wall synthesis [42]. Purine metabolism plays an integral role in cellular processes such as energy metabolism, cell signaling, and encoding the genetic make-up of all organisms [43]. Adenosine kinase (ADK) is a key enzyme in purine metabolism. It has been shown that the hormone FABP4 forms a new functional hormone complex with ADK and nucleoside diphosphate kinase (NDPK) to regulate extracellular ATP and ADP levels [44]. ATP-dependent chromatin remodeling complexes help reposition, assemble, migrate, and recombine nucleosomes, thereby changing the chromatin structure and adapting to the environmental response [45]. The chromatin complex subunit BAP18 contains a SWI3, ADA2, N-CoR, and TFIIIB (SANT) structural domain, which is present in subunits of chromatin remodeling complexes and plays a central role in chromatin remodeling by acting as a histone interaction module [46]. BAP18 directly interacts with DPY-30 and regulates the overall H3K4me3 level, suggesting a potential role for BAP18 in transcriptional regulation [47]. Proteomic studies of *Ericerus pela* have identified proteins that contribute to cold stress, such as cold acclimation proteins, glycerol biosynthesis-related enzymes, and heat shock proteins (HSPs) [48]. In addition, AQP knockdown was highly related to ubiquitin-mediated protein hydrolysis, nucleoplasmic transport, ribosomes, and endocytosis, which might reduce the cell access to water molecules from outside.

Specifically, AQPs play a key role in the cellular response to osmotic stress, and their dysfunction may lead to osmotic pressure imbalance, which interferes with normal biochemical reactions and metabolic processes within the cell, in turn affecting the accumulation of fat, glycerol, and protein [49]. The interplay between osmotic stress and cold survival has been widely recognized in many arthropods. *Ixodes scapularis* has significant physiological fluctuations in different life stages, and stage- or tissue-specific expression of osmoregulatory molecules reflects these needs. *IsAQP1* was highly expressed in salivary glands before feeding and was involved in osmotic regulation before or during feeding [32]. *Rhipicephalus microplus* accumulates fat, glycerol, and protein content after low-temperature domestication, and these substances work together to significantly improve their cold tolerance [50]. Thus, the inability to respond to osmotic stress may be an important reason for the suppression of cold tolerance.

Transcriptomic analyses suggest that cold tolerance in ticks may be influenced by regulation of transport, catabolism, and signal transduction. Knockdown of *HlAQP2* triggered the downregulation of genes mainly associated with the expression of insulin and cathepsin L in the autophagy pathway. The insulin signaling pathway in insects usually links metabolism and growth with the availability of nutrients, which is also a critical link in the regulation of diapause [51]. Cathepsin L belongs to the cysteine proteolytic enzyme of the papain family [52], which is involved in physiological processes such as hormone precursor processing and plays an important role in cell apoptosis [53, 54]. In the ladybird beetle *Coccinella septempunctata*, cathepsin L contributes to reproductive diapause by regulating lipid storage [55]. Therefore, it is speculated that downregulation of genes associated with insulin and cathepsin L might reduce the nutrient levels and heat production in ticks and decrease protease activity, resulting in lower tick survival in the cold.

Knockdown of *HlAQP3* caused downregulation of genes related to intracellular cholesterol transporter 1 (Niemann-Pick C1, NPC1) in the lysosome pathway. NPC1 is a multiplex transmembrane protein located in endocytosis and lysosomes and is mainly responsible for cholesterol transport in vivo [56, 57]. In mammals, NPC1 is abundant in intestinal epithelial cells and is responsible for steroid absorption [58]. In *Drosophila*, NPC1 is responsible for intracellular steroid transport and food steroid absorption [59, 60]. NPC1 has been found to regulate cholesterol transport and molting processes in *Bombyx mori* [61]. Knocking out the NPC1 gene affects the survival of *Bemisia tabaci* [62]. The lysosomal pathway was also identified in a transcriptomic study of the cold-tolerant species *Litopenaeus vannamei* [63]. Downregulation of the NPC1 in the lysosome pathway might affect physiological functions such as cholesterol absorption and transport in ticks, thus affecting the energy metabolism and survival rate of ticks at low temperatures.

The NF-kappa B signaling pathway was enriched in transcriptomic analyses in both the *HlAQP2* and *HlAQP3*-dsRNA injection groups. NF-kB refers to a family of transcription factors involved in the regulation of immune response, development, cell survival, and proliferation. They are effectors of signal transduction systems and can respond to many stimuli [64, 65]. Stimulation leads to rapid phosphorylation, ubiquitination, and eventual proteolytic degradation of IkappaB, resulting in the release of NF-kappaB, which translocates to the nucleus and activates the transcription of its target genes [66]. A close link between NF-kB and metabolism has also been suggested, with NF-kB regulating energy homeostasis through direct involvement in cellular networks that control glycolysis and respiration [67]. TNF receptor-associated factor 6 (TRAF6) is a RING E3 ligase that plays a key role in NF-kB activation induced by Toll-like receptor 4 (TLR4) signaling [68, 69]. Oligomerization of TRAF6 promotes auto-ubiquitination and catalyzes the synthesis of K63-linked polyubiquitin chains, which can be linked to TRAF6 itself or exist as free ubiquitin chains. Both ubiquitinated TRAF6 and free K63-linked polyubiquitin chains further activate downstream NF-kB pathways [70, 71]. Signaling through cell surface receptors, TRAF6, and the activation of nuclear factor NF-kB and mitogen-activated protein (MAP) kinase are essential for the survival and activation of all cells in the body [72]. TRAF6 expression was downregulated in this study, and it is speculated that the knockdown of AQP expression may have reduced the activation of downstream signals by TRAF6. The expression of AQPs may promote the activation of the downstream NF-kB pathway, which may improve cold tolerance.

**Fig. 6:**
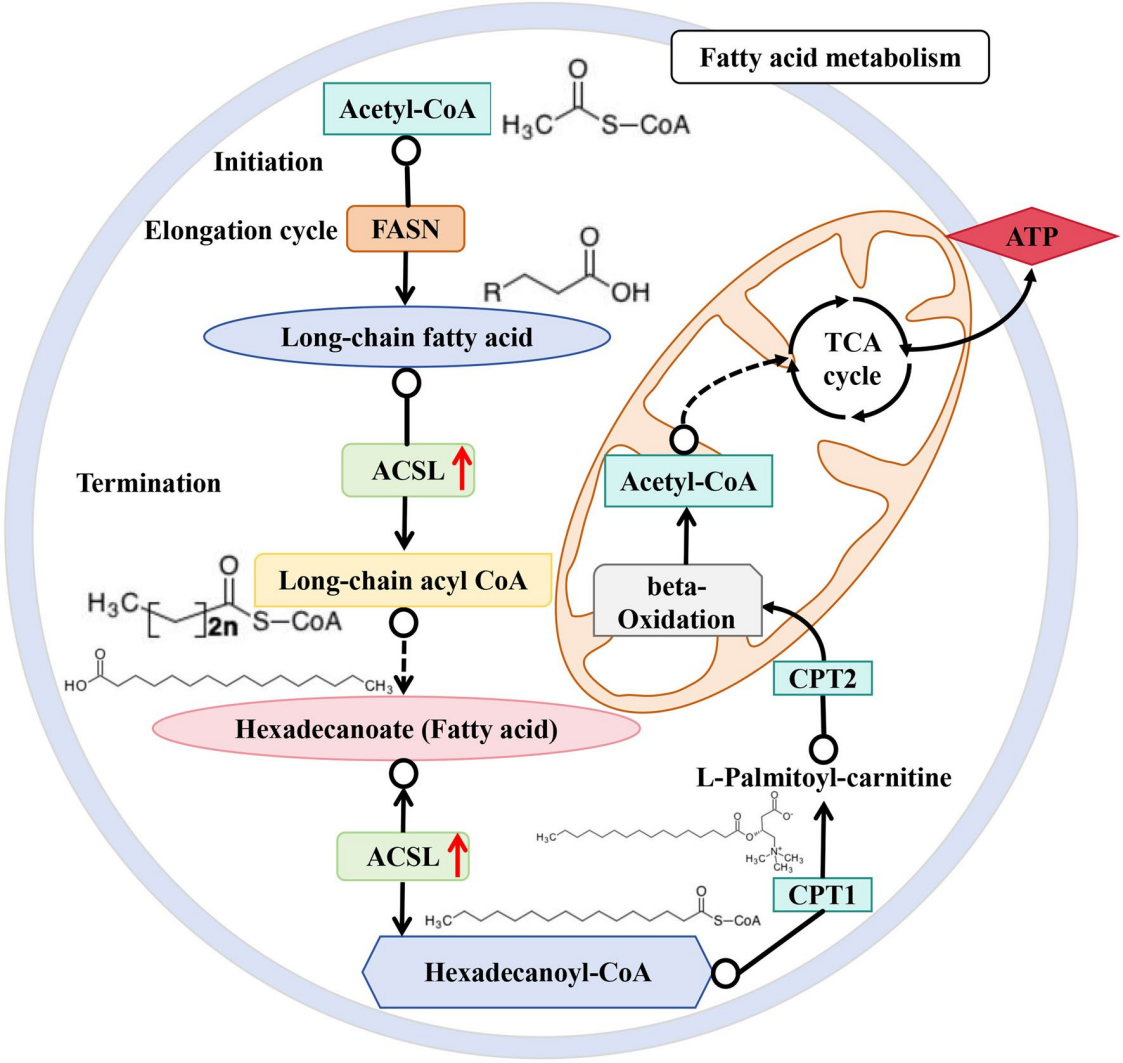
Schematic diagram of fatty acid metabolism modulated by aquaporins during cold response of *Haemphysalis longicornis* (red arrows indicate upregulation of the genes)

The fatty acid metabolism pathway was enriched through integrative transcriptomic and proteomic analyses, and ACSL expression was significantly upregulated. Fatty acid metabolism occurs in the mitochondrial matrix, where mitochondria, as the predominant catabolic organelles, catabolize carbon sources to produce energy through the fatty acid oxidation (FAO) and citrate (TCA) cycles [73]. Acetyl CoA can be used by fatty acid synthase (FASN) for the production of long-chain fatty acids or chain elongation of fatty acids [74]. ACSL catalyzes the initial step by converting free long-chain FA to acyl-coenzyme A [75], which is a key intermediate in lipid synthesis [76]. Fatty acids produced via fatty acid biosynthesis are metabolized by mitochondrial β-oxidation [77]. Cells preferentially access chemical energy stored in fatty acids (FAs) through β-oxidation in mitochondria (long, medium, and short chain FAs) [78]. A unique mitochondrial β-oxidation system provides an oxidative phosphorylation pathway for ATP synthesis [79]. Acetyl CoA from mitochondrial fatty acid β-oxidation is metabolized in the TCA cycle [80]. One of the main purposes of the TCA cycle is to utilize the chemical energy in acetyl coenzyme A to obtain the reducing power of NADH, which can then be used in a number of biological processes [81]. Cold tolerance in invertebrates is closely tied to membrane adaptation. *Enchytraeus albidus* phospholipid fatty acid composition varied significantly between populations and was correlated with population hardiness [82].Adjustments in membrane lipid composition, particularly fatty acid composition in insect membranes, affect membrane fluidity and integrity, thereby impacting insect survival at low temperatures [83]. Hence, modulation of cold stress by dynamic expression of AQPs in *H. longicornis* aquaporins might be highly associated with fatty acid metabolism (Fig. 6). However, the detailed underlying mechanism warrants further research.

## Conclusions

Three AQPs of *H. longicornis* were successfully characterized, and their involvement in the cold response of *H. longicornis* was confirmed. Integrative transcriptomic and proteomic analysis revealed that aquaporins could modulate cold stress through fatty acid metabolism and signal transduction by regulating cellular metabolism and protein homeostasis, which shed light on the molecular regulatory mechanisms involved in the cold response of ticks. However, the underlying mechanisms require further investigation.

## Supplementary Information

Additional file 1: Fig. S1. The relative expression of reference genes in *Haemphysalis longicornis* treated with low temperatures. A The relative expression of GAPDH. B The relative expression of tubulin. C The relative expression of β-actin.

Additional file 2: Fig. S2. The sequence characteristic of the aquaporins from *Haemphysalis longicornis*. A Schematic diagram of conserved domain of aquaporins (AQPs) in *H. longicornis*. B Prediction of secondary structure of AQPs in *H. longicornis*. C Prediction of tertiary structure of AQPs in *H. longicornis*. D Diagram of transmembrane region of AQPs in *H. longicornis.*

Additional file 3: Fig. S3. Phylogenetic and hydrophobicity analyis of aquaporins from *Haemphysalis longicornis*. A Phylogenetic tree of 3 AQPs and their homologous proteins from *H. longicornis*. A phylogenetic tree was constructed using neighbor-joining methods and visualized using iTOL. B Prediction of hydrophobicity of AQPs in *H. longicornis*.

Additional file 4: Fig. S4. Data independent acquisition proteomic analysis after knockdown of aquaporins in *Haemphysalis longicornis*. A Venn diagram analysis of intersecting proteins after AQP knockdown. B Enrichment analysis of differentially expressed protein GO after AQP knockdown in *H. longicornis.* BP: biological process; MF: molecular function; CC: cell component. **C** KEGG enrichment analysis of differentially expressed proteins after AQP knockdown in *H. longicornis.* UP: upregulated differential proteins; Down: downregulated differential proteins.

Additional file 5: Fig. S5. A GO enrichment analysis of differentially expressed genes of *HlAQP2* after RNAi in *Haemphysalis longicornis*. B GO enrichment analysis of differentially expressed genes of *HlAQP3* after RNAi in *H. longicornis*.

Additional file 6: Table S1. Primers for aquaporin of *Haemphysalis longicornis*.

Additional file 7: Table S2. Acetonitrile elution gradient setting and parameter settings for DIA.

Additional file 8: Table S3. The top 10 differentially expressed proteins after knockdown of *HlAQP2* in *Haemphysalis longicornis*. Table S4. The top 10 differentially expressed proteins after knockdown of *HlAQP3* in *Haemphysalis longicornis*. Table S5. The top 10 differentially expressed proteins after knockdown of *HlAQP5* in *Haemphysalis longicornis*.

Additional file 9: Table S6. The differentially expressed genes after knockdown of *HlAQP2* in *Haemphysalis longicornis*. Table S7. The differentially expressed genes after knockdown of *HlAQP3* in *Haemphysalis longicornis*.

## Data Availability

Sequences data that support the findings of this study have been deposited in the NCBI database under accession nos. OR754283–5. The mass spectrometry proteomics data have been deposited to the ProteomeXchange Consortium with the dataset identifier PXD058058. The transcriptomics raw data have been deposited into SRA of NCBI under PRJNA1186263.
