# Supplementary figures and images for: Aquaporins modulate the cold response of Haemaphysalis longicornis via changes in gene and protein expression of fatty acids
Source: Parasit Vectors. 2025 Feb 24;18:70. doi: 10.1186/s13071-025-06718-x (PMC11849292; doi:10.1186/s13071-025-06718-x)

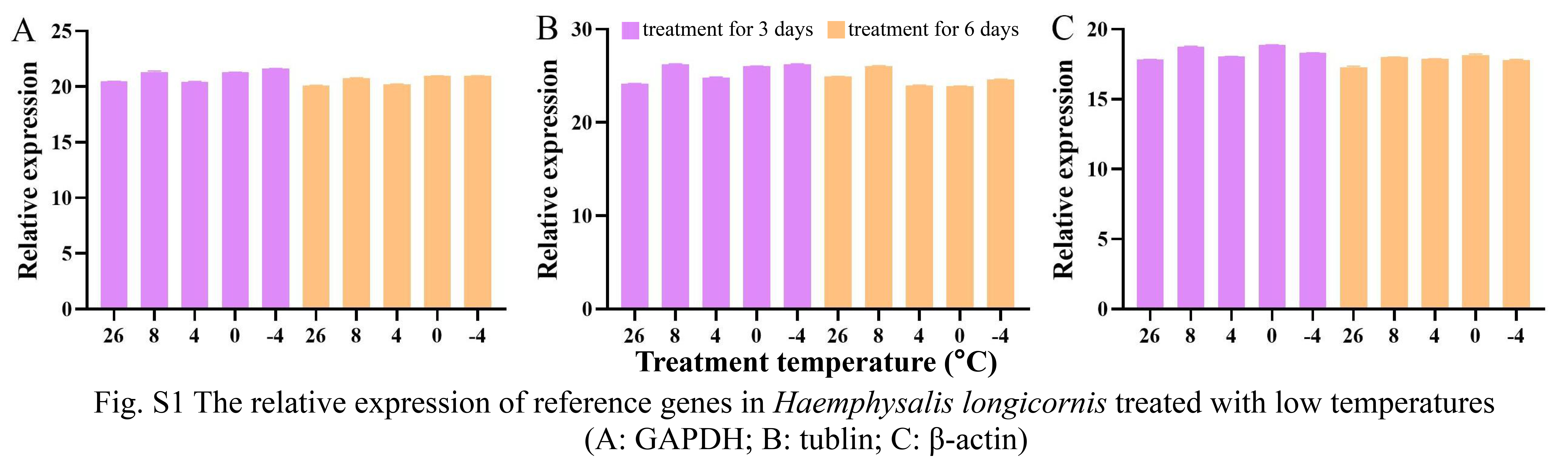

Supplement: Supplementary file 1 — Additional file 1: Fig. S1. The relative expression of reference genes in Haemphysalis longicornis treated with low temperatures. A The relative expression of GAPDH. B The relative expression of tubulin. C The relative expression of β-actin. [file 13071_2025_6718_MOESM1_ESM.jpg]

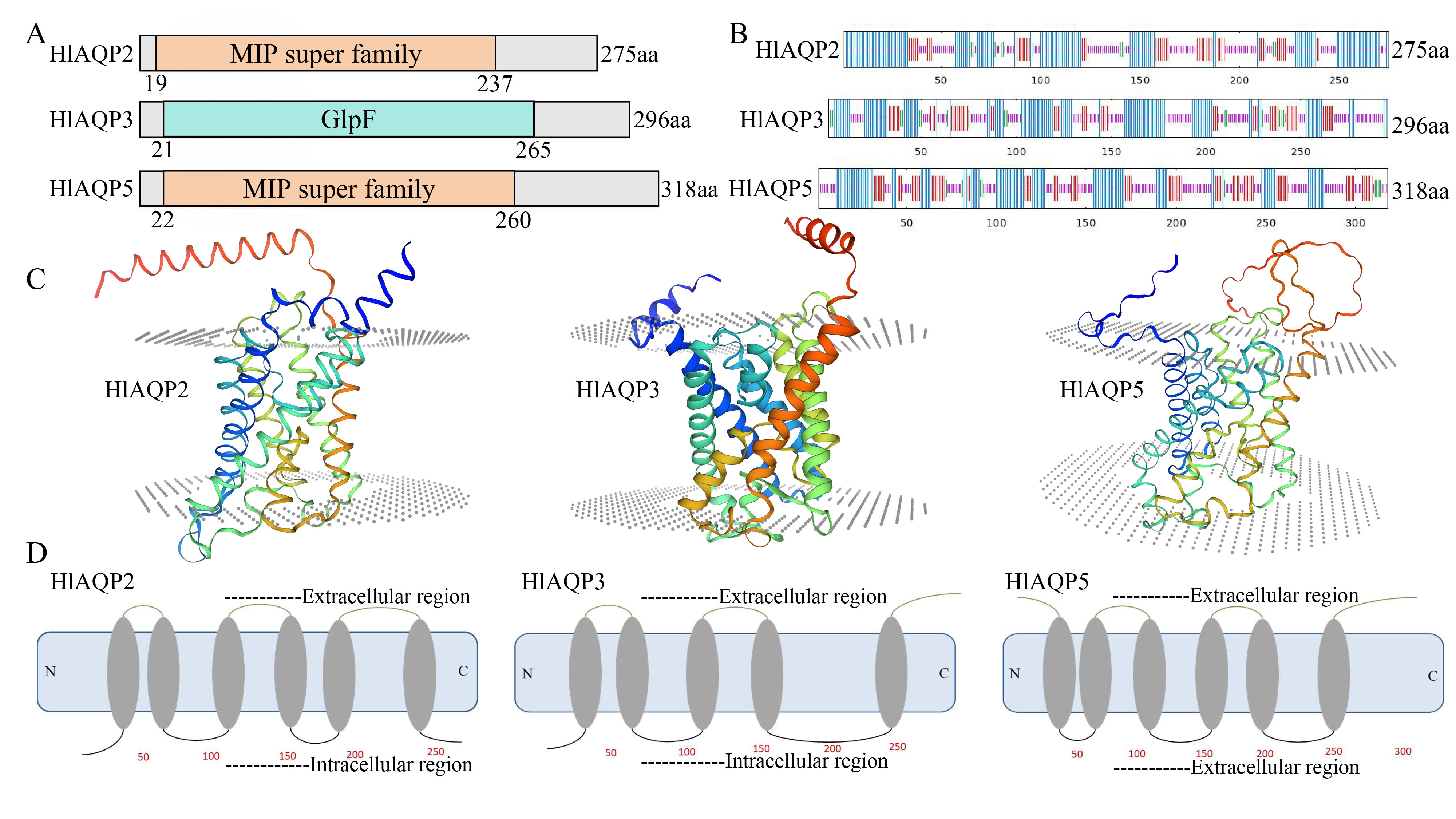

Supplement: Supplementary file 2 — Additional file 2: Fig. S2. The sequence characteristic of the aquaporins from Haemphysalis longicornis. A Schematic diagram of conserved domain of aquaporins (AQPs) in H. longicornis. B Prediction of secondary structure of AQPs in H. longicornis. C Prediction of tertiary structure of AQPs in H. longicornis. D Diagram of transmembrane region of AQPs in H. longicornis. [file 13071_2025_6718_MOESM2_ESM.jpg]

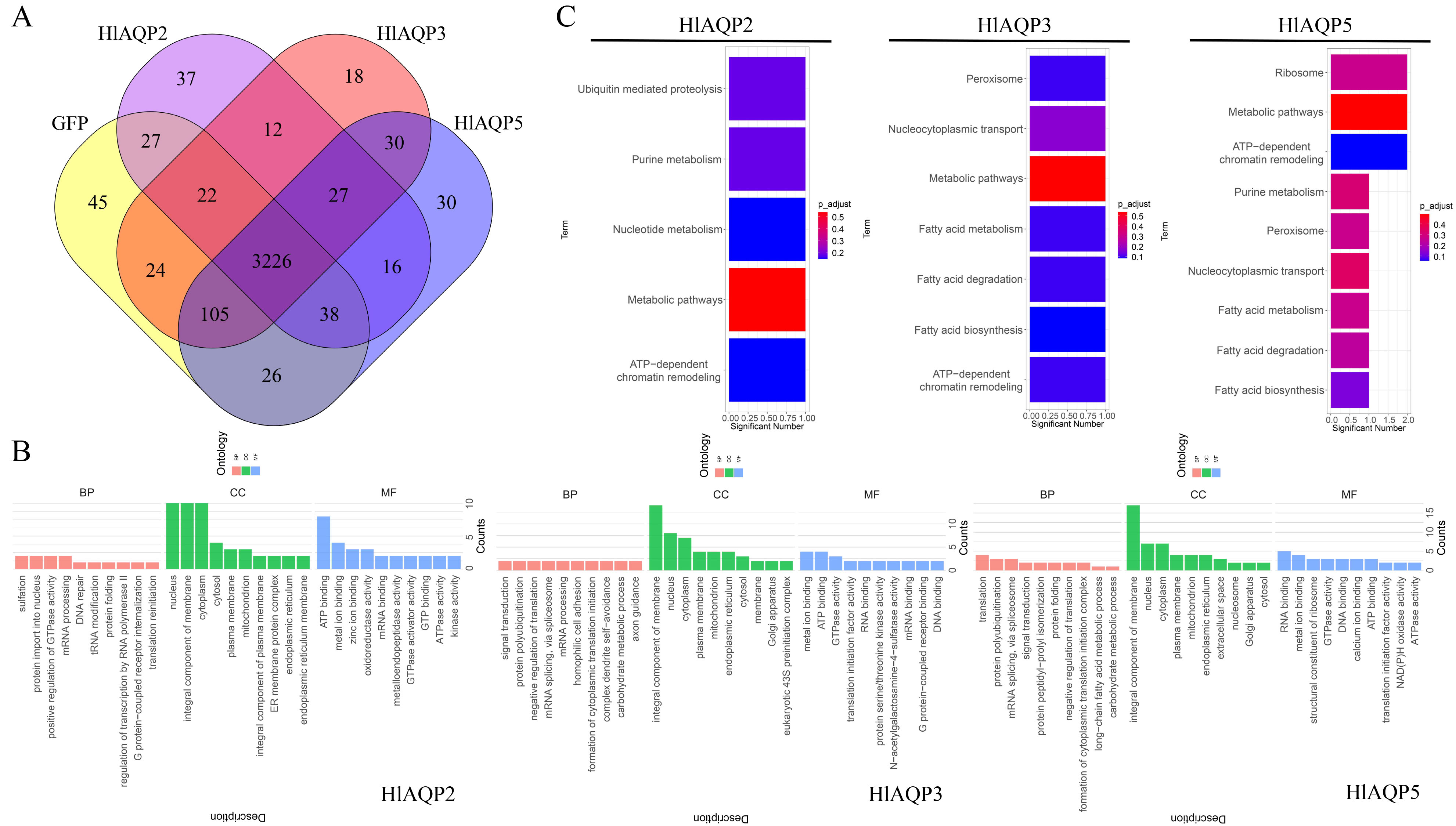

Supplement: Supplementary file 4 — Additional file 4: Fig. S4. Data independent acquisition proteomic analysis after knockdown of aquaporins in Haemphysalis longicornis. A Venn diagram analysis of intersecting proteins after AQP knockdown. B Enrichment analysis of differentially expressed protein GO after AQP knockdown in H. longicornis. BP: biological process; MF: molecular function; CC: cell component. C KEGG enrichment analysis of differentially expressed proteins after AQP knockdown in H. longicornis. UP: upregulated differential proteins; Down: downregulated differential proteins. [file 13071_2025_6718_MOESM4_ESM.jpg]

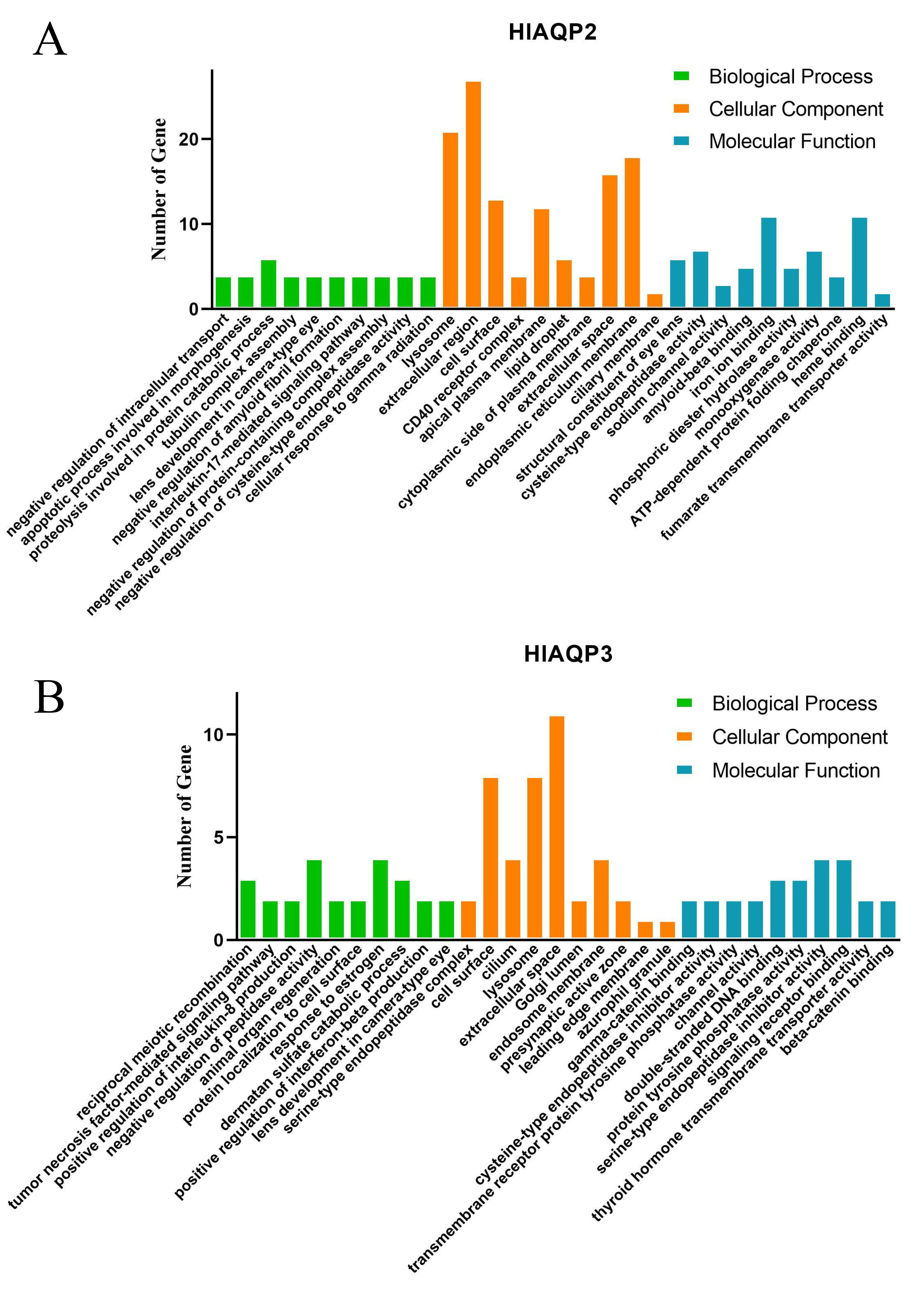

Supplement: Supplementary file 5 — Additional file 5: Fig. S5. A GO enrichment analysis of differentially expressed genes of HlAQP2 after RNAi in Haemphysalis longicornis. B GO enrichment analysis of differentially expressed genes of HlAQP3 after RNAi in H. longicornis. [file 13071_2025_6718_MOESM5_ESM.jpg]
