# Supplementary material for: Aquaporins modulate the cold response of Haemaphysalis longicornis via changes in gene and protein expression of fatty acids
Source: Parasit Vectors. 2025 Feb 24;18:70. doi: 10.1186/s13071-025-06718-x (PMC11849292; doi:10.1186/s13071-025-06718-x)
Supplement: Supplementary file 6 — Additional file 6: Table S1. Primers for aquaporin of Haemphysalis longicornis. [file 13071_2025_6718_MOESM6_ESM.docx]

**Table S1** Primers for aquaporins of *H. longicornis*

| **Test** | **Gene name** | **Primer sequence（5'-3'）** |
| --- | --- | --- |
| PCR | *HlAQP2* | F: GCCAGTTCCGCTCCTTT |
|  |  | R: TTAGATGGACGTAGTGCGG |
|  | *HlAQP3* | F: GCGTTCATCCGTGCCTG |
|  |  | R: GCCATTGGAGCCACGAG |
|  | *HlAQP5* | F: AACCCTCCGTTTCCTTTCT |
|  |  | R: CCTGTTGGCGTCCGTAA |
| qRT-PCR | *HlAQP2* | F: CATCGGCTTCGGCATCA |
|  |  | R: TGTAGAAGAAACTCCGCAGCA |
|  | *HlAQP3* | F: GCGTTCATCCGTGCCTG |
|  |  | R: CAAAACGCAGTCGCCTACC |
|  | *HlAQP5* | F: TTTGTGACCAAGAATGAAGCC |
|  |  | R: CAGCAGAAGGTCACAGCGAA |
| RNAi (+T7) | *HlAQP2* | F: GCTGCTGCGGAGTTTCTTC |
|  |  | R: ATGTCCTCGGTGGTGATGG |
|  | *HlAQP3* | F: CGAGAACGCTACCGCCC |
|  |  | R: GCCATTGGAGCCACGAGA |
|  | *HlAQP5* | F: GCTGGACAACTACGACGGC |
|  |  | R: CTGCGGCTTGGGGACAT |

Note: Addition of T7 promoter sequence to the 5' end of the specific primer (5' TAATACGACTCACTATAGG 3')
