# Supplementary material for: Aquaporins modulate the cold response of Haemaphysalis longicornis via changes in gene and protein expression of fatty acids
Source: Parasit Vectors. 2025 Feb 24;18:70. doi: 10.1186/s13071-025-06718-x (PMC11849292; doi:10.1186/s13071-025-06718-x)
Supplement: Supplementary file 7 — Additional file 7: Table S2. Acetonitrile elution gradient setting and parameter settings for DIA. [file 13071_2025_6718_MOESM7_ESM.docx]

**Table S2** Acetonitrile elution gradient setting and parameter settings for DIA

| **Acetonitrile elution gradient setting** | | | **Parameter settings (DIA)** | |
| --- | --- | --- | --- | --- |
| Time (min) | Flow（µL/min) | %B | Name of the Properties | Properties |
| 0.000 | 0.300 | 4.0 | Runtime | 0 to 60 min |
| 1.800 | 0.300 | 4.5 | Full-scan scan range | 400-1200 m/z |
| 2.000 | 0.300 | 5.0 | Full-scan Orbitrap Resolution | 120000 |
| 47.000 | 0.300 | 20.0 | Full-scan AGC Target | Custom |
| 57.000 | 0.300 | 35.0 | Full-scan Normalized AGC Target | 300% |
| 57.500 | 0.300 | 55.0 | Full-scan Maximum IT | 50 ms |
| 57.500 | Column Wash |  | HCD collision energy | 30% |
| 58.000 | 0.800 | 99.0 | DIA Window Type | Auto |
|  |  |  | Orbitrap Resolution | 30000 |
|  |  |  | AGC Target | Custom |
|  |  |  | Normalized AGC Target | 2000% |
|  |  |  | Maximum IT | Custom |
