# Supplementary material for: Aquaporins modulate the cold response of Haemaphysalis longicornis via changes in gene and protein expression of fatty acids
Source: Parasit Vectors. 2025 Feb 24;18:70. doi: 10.1186/s13071-025-06718-x (PMC11849292; doi:10.1186/s13071-025-06718-x)
Supplement: Supplementary file 8 — Additional file 8: Table S3. The top 10 differentially expressed proteins after knockdown of HlAQP2 in Haemphysalis longicornis. Table S4. The top 10 differentially expressed proteins after knockdown of HlAQP3 in Haemphysalis longicornis. Table S5. The top 10 differentially expressed proteins after knockdown of HlAQP5 in Haemphysalis longicornis. [file 13071_2025_6718_MOESM8_ESM.docx]

**Table S3** The top10 differentially expressed proteins after knockdown of *HlAQP2* in *H. longicornis*

| **Up-regulation of differential protein names** | **Fold change** | **Down-regulation of differential protein names** | **Fold change** |
| --- | --- | --- | --- |
| P15-2B protein | 26.11841032 | Poly (ADP-ribose) Glycohydrolase ARH3 | 0.000406 |
| Sodium/calcium exchanger | 23.94113621 | Cuticular protein | 0.000542 |
| Transmembrane protein 14C | 18.88165987 | Neprilysin-like | 0.000569 |
| Phosphatidylinositol transfer protein alpha isoform | 17.58222843 | Zinc finger CCCH Domain-containing protein 18-like isoform X1 | 0.000796 |
| Water-specific aquaporin | 16.46713 | Chitin binding peritrophin-A | 0.001947 |
| TATA box binding protein | 15.81868382 | MAP kinase-activated protein kinase | 0.002992 |
| Gephyrin isoform X2 | 13.19302601 | Eukaryotic translation initiation factor 4 gamma | 0.003601 |
| Uncharacterized protein LOC106463357 isoform X3 | 12.65401176 | Ankyrin-2-like isoform X7 | 0.003637 |
| Semaphorin-2A | 11.07656 | Salivary sulfotransferase | 0.008663 |
| Small nuclear ribonucleoprotein Sm D3 isoform X2 | 10.13751 | DnaJ homolog subfamily B member 9-like | 0.008863 |

**Table S4** The top10 differentially expressed proteins after knockdown of *HlAQP3* in *H. longicornis*

| **Up-regulation of differential protein names** | **Fold change** | **Down-regulation of differential protein names** | **Fold change** |
| --- | --- | --- | --- |
| Myosin XV | 22.95385 | Medium-chain acyl-CoA dehydrogenase | 0.001071 |
| TPA_inf: ixoderin precursor | 13.62521 | Cuticular protein | 0.00169 |
| Transposable element tc1 transposase | 8.687064 | RNA-binding protein 33-like | 0.002753 |
| Sdk-P1 | 8.22845 | Glutamine-dependent NAD(+) synthetase-like | 0.003793 |
| Nudix hydrolase | 7.057032 | Neprilysin-like | 0.00642231 |
| Enolase | 5.613286 | LIM domain-containing protein jub | 0.008231 |
| Cyclophilin | 5.310108 | Histone deacetylase 4, 5 | 0.009074 |
| Mucin-like protein | 5.095003 | MAP kinase-activated protein kinase | 0.011408 |
| Protein kinase | 5.001143 | Translocation protein SEC62 | 0.012773 |
| Lipid droplet-associated hydrolase-like | 4.136025 | Protein max-like isoform X2 | 0.013992 |

**Table S5** The top10 differentially expressed proteins after knockdown of *HlAQP5* in *H. longicornis*

| **Up-regulation of differential protein names** | **Fold change** | **Down-regulation of differential protein names** | **Fold change** |
| --- | --- | --- | --- |
| Myosin XV | 23.06464 | Cuticle protein | 0.000139 |
| Nudix hydrolase | 7.707662 | Asparaginase | 0.000572 |
| Transposable element tc1 transposase | 5.486831 | Secreted protein | 0.000683 |
| Serine protease inhibitor 6 RmS6 | 4.710293 | Medium-chain acyl-CoA dehydrogenase | 0.001267 |
| Vacuolar protein sorting-associated protein 37A-like | 4.475428 | Cuticular protein | 0.001547 |
| Cyclophilin | 4.103861 | RNA-binding protein 33-like | 0.001997 |
| Yolk cathepsin | 3.948974 | Glutamine-dependent NAD(+) synthetase-like | 0.002957 |
| Protein transport protein Sec24C-like | 3.563828 | Ixoderin precursor | 0.003587 |
| XAP-5 | 3.51172 | Tetraspanin | 0.004412 |
| Low-density lipoprotein receptor-like | 3.109811 | Ribosomal protein S15Aa | 0.010224682 |
