# Supplementary material for: Aquaporins modulate the cold response of Haemaphysalis longicornis via changes in gene and protein expression of fatty acids
Source: Parasit Vectors. 2025 Feb 24;18:70. doi: 10.1186/s13071-025-06718-x (PMC11849292; doi:10.1186/s13071-025-06718-x)
Supplement: Supplementary file 9 — Additional file 9: Table S6. The differentially expressed genes after knockdown of HlAQP2 in Haemphysalis longicornis. Table S7. The differentially expressed genes after knockdown of HlAQP3 in Haemphysalis longicornis. [file 13071_2025_6718_MOESM9_ESM.docx]

**Table S6** The differentially expressed genes after knockdown of *HlAQP2 in H. longicornis*

| **Gene name** | **log2 Fold change** | **Functional annotation** |
| --- | --- | --- |
| Glycerophosphocholine cholinephosphodiesterase ENPP6 | 4.434008 | Ether lipid metabolism |
| Plasma serine protease inhibitor | 4.006616 | Amoebiasis |
| Toll-like receptor 7 | 2.720710 | Toll and Imd signaling pathway |
| Elongation of very long chain fatty acids protein | 2.495666 | Fatty acid elongation |
| Heat shock protein 70 B2 | 2.215816 | MAPK signaling pathway |
| Arylsulfatase B | 1.591600 | Glycosaminoglycan degradation |
| Run domain Beclin-1-interacting and cysteine-rich domain-containing protein | 1.577272 | Autophagy - animal |
| (3R)-3-hydroxyacyl-CoA dehydrogenase | 1.510896 | Fatty acid biosynthesis |
| Alpha-crystallin B chain | 1.389229 | Protein processing in endoplasmic reticulum |
| Arylsulfatase B | 1.225210 | Lysosome |
| Protein 5NUC | -1.719668 | Purine metabolism |
| Retinol dehydrogenase 12 | -1.566163 | Retinol metabolism |
| Ornithine decarboxylase | -1.564494 | Arginine and proline metabolism |
| Cathepsin L | -1.558924 | Autophagy - animal |
| Juvenile hormone acid O-methyltransferase | -1.536313 | Insect hormone biosynthesis |
| Acetylcholinesterase | -1.524149 | Glycerophospholipid metabolism |
| Calcium-activated chloride channel regulator 3A-1 | -1.521262 | Renin secretion |
| Insulin-1 | -1.476028 | Autophagy - animal |
| TNF receptor-associated factor 6 | -1.385426 | NF-kappa B signaling pathway |
| Flavin-containing monooxygenase 5 | -1.299875 | Taurine and hypotaurine metabolism |

**Table S7** The differentially expressed genes after knockdown of *HlAQP3 in H. longicornis*

| **Gene name** | **log2 Fold change** | **Functional annotation** |
| --- | --- | --- |
| Glycerophosphocholine cholinephosphodiesterase ENPP6 | 3.454744 | Ether lipid metabolism |
| Alcohol dehydrogenase class-3 | 3.254389 | Fatty acid degradation |
| Toll-like receptor 7 | 3.080721 | Toll and Imd signaling pathway |
| Alpha-(1,3)-fucosyltransferase C | 2.361685 | Various types of N-glycan biosynthesis |
| Juvenile hormone acid O-methyltransferase | 2.097149 | Insect hormone biosynthesis |
| Toll-like receptor 13 | 1.483658 | Toll and Imd signaling pathway |
| Coagulation factor VII | 1.432016 | Complement and coagulation cascades |
| Probable chitinase 2 | 1.272778 | Amino sugar and nucleotide sugar metabolism |
| Juvenile hormone acid O-methyltransferase | 1.142836 | Insect hormone biosynthesis |
| Neurexin-4 | 1.057803 | Cell adhesion molecules |
| Casein kinase II subunit alpha | -1.203037 | NF-kappa B signaling pathway |
| Niemann-Pick C1 | -0.970875 | Lysosome |
| Glucose dehydrogenase | -0.749798 | Glycine, serine and threonine metabolism |
| Thromboxane-A synthase | -0.730702 | Arachidonic acid metabolism |
| Cytochrome P450 17A1 | -0.660854 | Steroid hormone biosynthesis |
| Putative protein heh-1 | -0.644135 | Cholesterol metabolism |
| Lactosylceramide 4-alpha-galactosyltransferase | -0.637915 | Glycosphingolipid biosynthesis |
| Adiponectin receptor protein | -0.631623 | AMPK signaling pathway |
| CTSB | -0.631528 | Autophagy |
| Multidrug resistance-associated protein 1 | -0.626261 | ABC transporters |
